# Supplementary figures and images for: THC reverses SIV-induced senescence in astrocytes: possible compensatory mechanism against HIV associated brain injury?
Source: Front Cell Neurosci. 2025 Sep 30;19:1642917. doi: 10.3389/fncel.2025.1642917 (PMC12521829; doi:10.3389/fncel.2025.1642917)

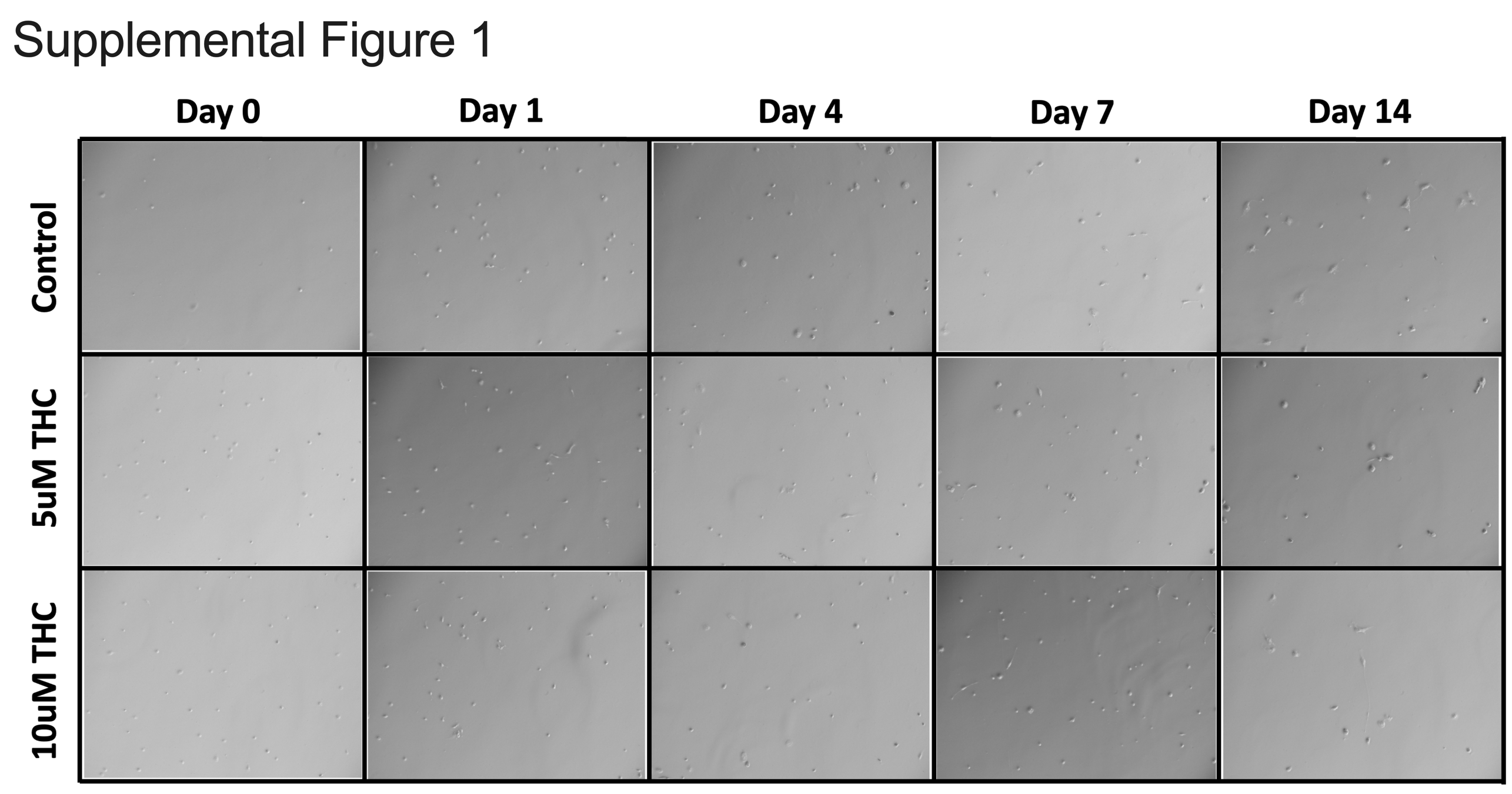

Supplement: Supplementary Figure 1 — Representative images of SIV+ mixed glial cultures treated with THC. Phase-contrast microscopy images of primary mixed glial cultures derived from SIV+ rhesus macaques following administration of 5 or 10 μM THC, compared to control conditions, over a 14 days period. Images were captured every 48 h to assess changes in cell number, density, morphology, and process formation over time. While the higher concentration of THC led to a progressive reduction in overall cell density (as observed in Figure 3), surviving glial cells exhibited enhanced stellation and process elongation: a possible shift toward a neuroprotective phenotype. This suggests that THC promotes cytoskeletal remodeling and astrocyte activation, potentially facilitating enhanced synaptic support and immune modulation despite an overall reduction in cell viability. Notably, in the 10 μM THC condition, glial cells demonstrated the most pronounced process elongation and branching complexity, possibly compensating for the decline in total cell numbers. These findings highlight the dual role of THC in modulating glial survival and function, where higher THC concentrations may induce cellular stress leading to cell loss, yet simultaneously promote a more complex and supportive astrocytic network. [file Image_1.tiff]
